# Profile, Outcomes, and Determinants of Unsuccessful Tuberculosis Treatment Outcomes among HIV-Infected Tuberculosis Patients in a Nigerian State

**DOI:** 10.1155/2014/202983

**Published:** 2014-11-16

**Authors:** Daniel Chukwunweolu Oshi, Sarah Nakalema Oshi, Isaac Alobu, Kingsley Nnanna Ukwaja

**Affiliations:** ^1^Centre for Development and Reproductive Health, Enugu, Enugu State 400001, Nigeria; ^2^National Tuberculosis and Leprosy Control Programme, Ministry of Health, Abakaliki, Ebonyi State 480001, Nigeria; ^3^Department of Internal Medicine, Federal Teaching Hospital, Abakaliki, Ebonyi State 480281, Nigeria

## Abstract

*Background*. Few studies have evaluated the rate of tuberculosis (TB)/human immunodeficiency virus (HIV) coinfection and the determinants of its treatment outcomes in Africa. We aimed to determine the predictors of unsuccessful treatment outcomes in HIV-infected tuberculosis patients in Nigeria. *Methods*. A retrospective cohort study design was used to assess adult TB/HIV patients who registered for TB treatment in two health facilities in Ebonyi State, Southeast Nigeria, between January 2011 and December 2012. Predictors of unsuccessful treatment outcomes were determined using multivariable logistic regression analysis. *Results*. Of 1668 TB patients, 342 (20.5%) were HIV coinfected. Of these, 195 (57%) had smear-negative pulmonary TB and 11 (3.2%) had extrapulmonary TB. Overall, 225 (65.8%) patients achieved successful outcomes, while 117 (34.2%) had unsuccessful outcomes. The unsuccessful treatment outcomes were due to “default” (9.9%), “death” (19%), “treatment failure” (1.5%), and “transferring out” (3.8%). Independent determinants for unsuccessful outcomes were receiving care at a public facility and noninitiation of antiretroviral therapy. *Conclusion*. There is need for the reevaluation of the quality of public sector treatment services provided for TB/HIV patients as well as further expansion of TB/HIV collaborative activities in rural areas, and interventions to reduce mortality and default rates among TB/HIV patients are urgently needed in Nigeria.

## 1. Introduction

Tuberculosis (TB) is the commonest opportunistic infection and the most important cause of morbidity and mortality in human immunodeficiency virus- (HIV-) infected patients [1]. Also, HIV is the greatest predictor for the progression of latent TB infection to TB disease, thus fuelling the TB epidemic in many countries, especially those in sub-Saharan Africa [1, 2]. Nigeria ranks tenth among the 22 high TB burden countries with 97,853 patients notified in 2012 accounting for an estimated prevalence rate of 161 per 100,000 population [2, 3]. Although Nigeria has met the World Health Organization (WHO)/millennium development goals framework (2011–2015) TB treatment success rate indicator of 85% for all TB cases in 2011, estimated case detection rate remains low (51%) in 2012 [3, 4]. Although final results of a recent national survey are still being awaited, preliminary data showed a high prevalence of TB not notified to the National TB Control Programme (NTP) in Nigeria [3]. Also, Nigeria has the second largest burden of HIV in Africa with about 3.1 million people (4.1% of the population) estimated to be living with HIV in 2010 and, with a national HIV coinfection rate of 23% among TB patients, HIV is believed to be driving this epidemic [3, 5].

Between 2000 and 2009, the estimated prevalence rate of TB in Ebonyi State fell from 123 per 100,000 population to 77 per 100,000 population [6]. However, the estimated TB case detection rate during the period rose from 27% in 1999 to around 40% during 2007 to 2009 [7]. Also, HIV prevalence assessed through national sentinel surveys among pregnant women showed that HIV prevalence in Ebonyi State has declined from 9.3% (1999) to 3.3% (2010) [5], but TB/HIV coinfection rate was 28% [8]. In Sub-Saharan Africa, unlike Asia [9–13], Europe [14, 15], and South America [16], few studies have evaluated the TB treatment outcomes of HIV-infected TB patients [17–19]. The available studies in Nigeria were conducted on TB/HIV patients managed during the early periods of TB/HIV collaborative activities when they are eligible for commencement of antiretroviral therapy (ART) within two months of TB treatment only if their CD4+ count fell below 200 cells/mL [8, 20–23], and HIV-infected TB patients were treated for TB at the TB clinics. Thus, TB/HIV patients faced logistic problems in keeping different appointment dates for both the HIV and TB clinics. HIV-infected TB patients managed during this period were found to have high TB treatment default rates of up to 30% and case fatality rates of 6.6–15.5% in Nigeria [8, 20–22].

With the ongoing expansion of TB/HIV collaborative activities where HIV-infected TB patients receive anti-TB and ART from the same clinic and the adoption of the recent WHO recommendation that ART be initiated for all TB-HIV patients irrespective of their CD4+ count by Nigeria [2, 23, 24], there is a need to reassess the treatment outcomes of HIV-infected TB patients. Furthermore, studies in other settings have identified several determinants of unsuccessful treatment outcomes among HIV-infected TB patients, including not receiving cotrimoxazole therapy, adverse event during treatment, pulmonary TB case, retreatment TB case, noninitiation of ART, intravenous drug use, gender, lymphadenopathy, low serum albumin, and CD4+ count below 200 cells/mL [9–11, 13, 14, 16]. However, only few studies have evaluated the determinants of unsuccessful outcomes among TB/HIV patients in sub-Saharan Africa [19], and none in Nigeria. Since these determinants can vary between different populations and health systems, it is important to evaluate these factors in specific settings in order to inform the development of targeted interventions.

The aim of this study was to describe the epidemiological characteristics, treatment outcomes, and the determinants of unsuccessful outcomes among HIV-infected TB patients in a resource-poor high TB/HIV incidence African setting. Specific objectives were to determine among adult TB patients in Nigeria in 2011 and 2012 the following: (i) the rate of HIV coinfection and the profile of HIV-infected TB patients, (ii) TB treatment outcomes of TB/HIV patients, and (iii) the predictors for unsuccessful treatment outcomes among HIV-infected TB patients.

## 2. Materials and Methods

### 2.1. Study Design

This was a retrospective cohort study of adult (≥15 years) HIV-infected TB patients treated in two hospitals (one public/tertiary care urban and one private/not-for-profit/mission secondary care rural hospital) between 1 January 2011 and 31 December 2012 in Ebonyi State, Nigeria.

### 2.2. Study Setting

Ebonyi is one of Nigeria's 36 states. It is located in Southeastern Nigeria with 73.6% of its 2.5 million population living below the poverty line and 75% of its inhabitants living in rural areas [25, 26]. Ebonyi has 13 local government areas (LGAs) with about 130 health care facilities currently providing directly observed treatment short course (DOTS) services for TB [6]. All treatment units have standard unit registers from the NTP and a TB-focal person responsible for coordination of all TB-related activities in the unit. Each LGA has a tuberculosis control (TBC) supervisor responsible for managing and coordinating TB control activities as well as keeping up-to-date and accurate record of activities [2, 6]. They also provide monthly report to the State TB programme officer whose responsibilities among others include collection, collation, and analysis of data on TBC activities in the state, monthly visitations to the LGA TB facilities for supervision, coordination of quarterly report meetings with LGA TBC supervisors, and dissemination of reports to the Federal Ministry of Health as well as other organizations as appropriate. Nonprofit mission hospitals are prominent in rural Ebonyi State because functional public sector primary and secondary healthcare facilities are often lacking. The two hospitals used for this study account for about 50% of annual TB case notification in the state and they serve an estimated 1.5 million people yearly [6].

### 2.3. Diagnosis and Treatment of TB and HIV

Adult patients having a cough lasting for two weeks or more who passively sought for care at the study facilities were clinically suspected of having TB and were investigated for TB by providing three sputum samples for acid-fast bacilli (AFB) microscopy [2]. Individuals with at least one positive sputum smear for AFB are registered as smear-positive pulmonary TB. Patients with smear negative sputum do a chest X-ray. Clinical assessments, nonresponse to empirical antibiotics treatment for pneumonia, and chest X-ray findings are used in making a diagnosis of smear negative TB by a clinician. Extrapulmonary TB is diagnosed through clinical assessments and tissue histology (if available) [23, 27]. All adults suspected of having TB undergo HIV counselling and testing at the time of submission of sputum for AFB/laboratory investigation for TB [2, 23, 27].

In accordance with the current WHO and national guidelines [2, 23, 27], the NTP in Ebonyi State phased out the 8-month anti-TB regimen (consisting of 2 months of rifampicin [R], isoniazid [H], pyrazinamide [Z], and ethambutol [E]/then 6 months of ethambutol and isoniazid [2RHZE/6EH] in the 2011 cohort [2]). All adult TB patients diagnosed in 2012 were treated using the 6-month anti-TB regimen consisting of 2-month RHZE/and 4-month RH [2RHZE/4RH]. Patients undergoing retreatment receive a 3-month intensive phase with the addition of streptomycin to the above four drugs for the first two months [2, 23, 27], and anti-TB treatment in patients with extrapulmonary TB may be extended to 12 months. All TB patients were treated using the community-based DOTS. Community-based DOTS requires that a family/community member (DOTS-supporter) monitors the patients' daily intake of the anti-TB drugs. The activities of the DOTS-supporter are monitored by a volunteer community health extension worker (CHEW) living in the patient's community. LGA TBC supervisors monitor the activities of the CHEWs in their area. All TB/HIV patients are offered trimethoprim/sulfamethoxazole (cotrimoxazole preventive therapy [CPT]) to prevent other opportunistic infections. Current HIV treatment follows national/WHO guidelines with ART initiated between two weeks and two months of commencing anti-TB treatment [2, 23, 27]. The commonly administered ART regimen is efavirenz, zidovudine, and lamivudine.

### 2.4. Sample Size

With a sample of 322 patients, we were able to detect an expected rate of unsuccessful outcomes of 47.6% among TB/HIV patients at 95% confidence level and an absolute precision of 5% (Statcalc in Epi Info) [8, 28]. Furthermore, in line with theoretical assumptions and guidelines of multiple regression, this sample size would be stable allowing the analysis of 15 predictor variables [28].

### 2.5. Data Collection and Analysis

Using a standardized form, we retrieved data about all HIV-infected TB patients treated at the participating centres from the TB treatment registers. Information collected included registration status, age, gender, residence, smear/disease status, ART/CPT use, and treatment outcome. We used the standard WHO definitions for TB disease classification, registration, and treatment outcome categories (cured, completed treatment, failure, defaulted treatment, died, and transferred out) [2, 23, 27]. TB registration status was divided into “new” or “previously treated,” and treatment outcome was divided into “successful” or “unsuccessful.” A successful outcome includes TB/HIV infected patients who were cured* (a patient who was smear-positive at diagnosis, who completed 6 or 8 months of treatment, and who was smear-negative at the end of 6th month or 7th month of treatment and at least one previous occasion)* or completed treatment* (any patient who was smear-positive at diagnosis and who completed treatment but in whom smear examination results are not available at the end of treatment or all smear-negative and extrapulmonary patients who completed treatment)* [2, 23, 27].

Statistical analyses were performed using Epi Info (Epi Info version 3.4.1; Centers for Disease Control, Atlanta, GA, USA). Patient characteristics were described using proportions. Continuous variable(s) were summarised using mean ± standard deviation. Categorical groups' comparisons were performed using the chi-square tests. In univariate analysis, we determined demographic and clinical factors associated with an unsuccessful outcome. Crude odds ratios (ORs) and adjusted ORs with their 95% confidence intervals (CIs) were estimated using multivariable logistic regression analysis with “unsuccessful treatment outcome” as the outcome variable. First, we performed a univariate analysis, and then a stratified analysis was performed to determine the occurrence of interaction and confounding between predictor variables. Interactions were determined by chi-square for differing ORs by stratum, while confounding was assessed by comparing the Mantel-Haenszel summary OR to the crude OR. Then, a multivariable logistic regression analysis was performed; we selected variables for inclusion based on clinical importance or a *P* value < 0.2 in the univariate analysis. Variables were also checked for colinearity and were model fit assessed with the likelihood ratio test.

## 3. Results

From 1 January 2011 to 31 December 2012, 1668 TB patients were registered for treatment in the two study health facilities. Of these, 342 (20.5%) were found to be HIV-positive. There were 178 (52%) males and 164 (48%) females; their mean (standard deviation) age was 36.3 (10.5) years and 233 (68.1%) lived in a rural area (Table 1). Also, 331 (96.8%) of the patients had pulmonary tuberculosis (with 195 (57%) having smear-negative and 136 (39.8%) having smear-positive pulmonary TB), and 319 (93.3%) were registered as a new TB case (Table 1).

Successful treatment outcomes were achieved by 225 (65.8%) of the patients (with 26% of patients cured and another 39.8% classified as those who completed treatment), and 117 (34.2%) had unsuccessful outcomes. The unsuccessful treatment outcomes were due to those who “defaulted” (9.9%; *n* = 34), “died” (19%; *n* = 65), “failed treatment” (1.5%; *n* = 5), and “transferred out” (3.8%; *n* = 13).

The treatment outcomes of TB/HIV patients stratified by demographic and clinical characteristics are as shown (Table 1). The proportion of patients with successful outcomes was significantly associated with residence (rural versus urban; 60.9% versus 76.1%; *P* = 0.006) and facility where care was given (public versus private; 48.4% versus 72.1%; *P* < 0.001). However, age group (*P* = 0.75), gender (*P* = 0.50), type of TB (*P* = 0.42), registration status (*P* = 0.95), regimen received (*P* = 0.11), use of ART (*P* = 0.81), and use of CPT (*P* = 0.94) were not significantly associated with treatment outcomes (Table 1).

A stratified analysis showed significant interaction between patients' residence and facility where care was given and their treatment outcomes. Thus, we performed two univariate and multivariable logistic regression analyses according to residence (rural and urban) to identify predictors for unsuccessful outcomes (Tables 2 and 3). Among rural TB/HIV patients (Table 2), only receiving care at a public facility was significantly associated with unsuccessful treatment outcomes (*P* < 0.001). After adjusting for potential confounders, receiving care at a public facility remained an independent predictor for unsuccessful outcomes (aOR 3.8; 95% confidence interval (CI) 1.9–7.7). Among urban TB/HIV patients (Table 3), age, gender, facility where care was given, treatment category, anti-TB regimen received, and use of ART or CPT were not significantly associated with unsuccessful outcomes in univariate analysis. After adjusting for confounders, receiving care at a public facility (aOR 4.5; CI 1.2–16.7) and not receiving ART (aOR 7.0; CI 1.3–36.8) were independent predictors for unsuccessful outcomes.

## 4. Discussion

In this study, we found that the 20.5% (344) of TB patients were coinfected with HIV during the study period, and 65.8% of them had a successful treatment outcome. Also, we found that majority of the patients who had unsuccessful outcomes either died or defaulted from treatment. Furthermore, receiving care at the public facility predicted the occurrence of unsuccessful outcomes among patients residing in a rural area, while receiving care at the public facility and not receiving ART predicted the occurrence of unsuccessful outcomes among TB/HIV patients residing in an urban area.

The prevalence of TB/HIV coinfection observed in this study was similar to the current national prevalence of 23% in Nigeria [3]. Previous studies in the country have shown that HIV infection in TB patients ranged between 28% and 43% [8, 21, 22]; it may be that the prevalence of HIV in TB patients in Nigeria is decreasing. Our result was consistent with findings from Togo (23.7%) but was lower than the prevalence of 35.2% and 56% observed in Cameroon and Malawi, respectively [17–19]. Compared to lower HIV incidence settings, the prevalence of HIV infection in TB was 11.8% in Malaysia, 12.4% in Spain, and 2.9% in Vietnam [9, 12, 13]. Thus, TB/HIV coinfection is a major challenge in Africa that needs to be controlled.

Also, we found that HIV-infected TB patients in Ebonyi State have poor treatment outcomes with a treatment success rate of 65.8%, well below the 85% target recommended by the WHO for all TB cases [3, 4]. Several studies have found that HIV-infected TB patients have lower treatment success rate than HIV-negative patients [13–16, 21]. The proportion of patients with a successful treatment outcome in this study agrees with other findings from Nigeria (60.3%), Togo (64.3%), Cameroon (69%), Vietnam (71%), and India (77%) [9, 11, 17, 18, 21] but was higher than the treatment success rate of 53.4%% documented in a low-incidence setting [13]. Our finding suggests that there is a need for strategies to improve treatment outcomes among HIV-infected TB patients in TB/HIV-collaborative activities.

Only few studies have evaluated risk factors for unsuccessful outcomes specifically for HIV-infected TB patients in Africa [19]. We found that rural resident TB/HIV persons who were treated at the public healthcare facility were more likely to have unsuccessful TB treatment outcomes. This may be because TB/HIV patients residing in rural areas face geographic and economic barriers in accessing the public sector health services [29]. Our finding suggests the need for further expansion of TB/HIV collaborative activities in rural areas of Nigeria. Also, we found that urban resident TB/HIV persons who received care at the public (tertiary) hospital had higher risks of achieving unsuccessful TB treatment outcomes. The reason may be because most TB/HIV persons seen in the hospital may be sicker than those seen at the secondary health facility; thus, comorbidities may be responsible for the increased risk of unsuccessful outcomes among TB/HIV patients seen at the public hospital. Another probable reason for the poorer treatment outcomes at the public facility may be from a lower quality of TB/HIV services offered. However, all TB/HIV patients irrespective of where they are managed receive quality services in Nigeria as the NTP undertake monthly monitoring/field supervisory visits at the health facility level to ensure compliance with national TB/HIV treatment guidelines [2, 23, 24]. Further studies are needed to understand whether these differences in mortality and default are real and, if they are, to identify strategies for responding to this higher risk of poor outcomes among TB/HIV patients in public healthcare facilities in Nigeria.

Previous studies reported an improvement in successful TB treatment outcomes among HIV-infected TB patients prescribed ART [10, 13, 16, 19]. This was consistent with the finding in this study among urban TB/HIV patients. However, there was no relationship between ART use and treatment outcomes among HIV-infected patients residing in a rural area. This may be because despite the well-documented benefits of ART in TB/HIV patients and the increased availability of ART in Nigeria, only one-third of rural TB/HIV patients were prescribed ART. The low uptake of ART among rural TB/HIV patients in this study may have been responsible for the lack of association observed. The current national and WHO guidelines leave the decision on when to start ART in HIV-infected TB patients to be decided by the managing physician [23]. The commencement of ART may be deferred until the end of tuberculosis therapy due to concerns about potential drug interactions between rifampicin and antiretroviral drugs, the immune reconstitution inflammatory syndrome, overlapping side effects, taking large quantity of pills for TB/HIV, and patients readiness to maintain ART and anti-TB treatment once started [13]. More than half of the patients in this study received CPT. Despite documented benefits [9], we found no relationship between CPT use and successful treatment outcomes. This may due to poor patients' compliance with the prescribed CPT; however, we are unable to confirm this from the data in the TB treatment registers. CPT uptake among TB/HIV patients needs to be further improved and further studies on patients' compliance with CPT and its impact on treatment outcomes are needed in our setting.

This study has shown that a fifth of HIV-infected TB patients died during treatment. The high mortality rate observed is consistent with data from other developing countries [8–11, 13, 14, 17, 18, 21, 22]. The reasons for high mortality rate of HIV-infected TB patients are not clear. Late presentation and diagnosis of HIV in these patients may be a cause for the high mortality rate. Also, for unclear reasons, HIV-infected smear-negative pulmonary TB patients in sub-Saharan Africa have been found to have a higher mortality than other forms of TB [30]. This may also be implicated in the high mortality rate observed. There is a need for prospective studies of HIV-infected TB patients in order to better understand factors associated with mortality in these patients.

Further, we have also shown that a tenth of TB/HIV coinfected persons currently default from treatment. This finding was much lower than a default rate of 30% previously observed in the setting [8, 20]. This suggests that the health education, enhancement of TB/HIV collaborative activities, and defaulter tracing interventions instituted by the NTP following the previous report in the state have largely been successful, although there is room for further improvement. Our finding was higher than a default rate of 1% found in Vietnam but was very much lower than a default rate of 16% in India, 25.6% in Malaysia, and 28.7% in Cameroon among TB/HIV patients [10, 13, 18]. Our study suggests that additional strategies are needed to sustain low default rates among TB/HIV patients because defaulters could be the source of persisting community transmission of TB including potentially drug resistant TB.

There are some limitations in this study. The study was retrospective using data that were recorded in TB registers. We could not independently verify the accuracy of these records nor could we collect additional data needed to confirm or refute our findings. However, since our findings were based on routine surveillance data, it reflects operational reality. Also, the study was conducted among rural and urban patients in Ebonyi State; it may not reflect observations in other regions in Nigeria. However, our sample was representative of Ebonyi State and Nigeria with respect to rural residence and the prevalence of HIV-TB coinfection, reducing concerns that the patients studied were systematically different from the population in Nigeria. We do not have information on CD4+ count, opportunistic infections, diarrheal disease, ART regimen, timing of ART, ART/CPT drug compliance, and drug adverse effects; these factors may positively/negatively affect TB treatment outcomes. The national TB treatment registers need to be modified to accommodate some of these data for HIV-infected TB patients. A further prospective study including these data would improve upon these limitations. Furthermore, resistance to anti-TB drugs may have been responsible for the high rates of unsuccessful outcomes observed. Routine culture and anti-TB drug sensitivity testing were not performed for all HIV-infected TB patients. Therefore, we are unable to report the burden of ant-TB drug resistance in TB/HIV patients in our setting. However, with the recent expansion of the Xpert (GeneXpert) MTB/RIF molecular testing techniques currently being proposed to be used for special populations like TB/HIV patients by the NTP [31], the burden of rifampicin-resistant TB in TB/HIV patients in Nigeria may soon be adequately documented.

## 5. Conclusion

Receiving care at a public facility and noninitiation of ART were risk factors for unsuccessful TB treatment outcomes among HIV-infected TB patients. We recommend the following: (i) the expansion of TB/HIV collaborative activities in private and public facilities especially in rural and remote settings of Ebonyi State and Nigeria, (ii) all TB/HIV patients be initiated on ART within two months of starting TB treatment in line with the current national/WHO guidelines, (iii) HIV-infected TB patients receiving care especially in public facilities be closely monitored during treatment, (iv) the ongoing efforts to improve retention of care among TB/HIV coinfected patients be sustained, and (v) prospective studies on factors associated with mortality and interventions effective at reducing them for HIV-infected TB patients be carried out in Nigeria.

## Figures and Tables

**Table 1:** Sociodemographic and clinical characteristics of 342 TB/HIV coinfected patients in Ebonyi State, Nigeria, stratified by tuberculosis treatment outcome.

Characteristics	Frequency N (%)	Successful outcome n (%)	Unsuccessful outcome n (%)	*P* value
Age (years)				0.75
≤40	255 (74.6)	169 (66.3)	86 (33.7)	
>40	87 (25.4)	56 (64.4)	31 (35.6)	
Gender				0.50
Female	164 (48.0)	105 (64.0)	59 (36.0)	
Male	178 (52.0)	120 (67.4)	58 (32.6)	
Residence				0.006
Urban	233 (68.1)	83 (76.1)	26 (23.9)	
Rural	109 (31.9)	142 (60.9)	91 (39.1)	
Facility				<0.001
Private	251 (73.4)	181 (72.1)	70 (27.9)	
Public	91 (26.6)	44 (48.4)	47 (51.6)	
Type of TB				0.42
Pulmonary	331 (96.8)	219 (66.2)	122 (33.8)	
Extrapulmonary	11 (3.2)	6 (54.5)	5 (45.5)	
Treatment category				0.95
New	319 (93.3)	210 (65.8)	109 (34.2)	
Previously treated	23 (6.7)	15 (65.0)	8 (35.0)	
TB regimen received				0.11
Regimen 1	196 (57.3)	122 (62.2)	74 (37.8)	
Regimen 2	146 (42.7)	103 (70.5)	43 (29.5)	
Received ART				0.81
Yes	117 (34.2)	78 (66.7)	39 (33.3)	
No	225 (65.8)	147 (65.3)	78 (34.7)	
Received CPT				0.97
Yes	189 (55.3)	124 (65.6)	65 (34.4)	
No	153 (44.7)	101 (66.0)	52 (34.0)	

Regimen 1: 2RHZE/6EH; regimen 2: 2RHZE/4RH (R: rifampicin, H: isoniazid, Z: pyrazinamide, and E: ethambutol); ART: antiretroviral therapy; TB: tuberculosis; HIV: human immunodeficiency virus; CPT: cotrimoxazole preventive therapy.

**Table 2:** Predictors of unsuccessful tuberculosis treatment outcomes among 233 rural TB/HIV patients in Ebonyi State, Nigeria, 2011-2012.

Characteristics	Successful outcome n (%)	Unsuccessful outcome n (%)	Crude OR (95% CI)	Adjusted OR (95% CI)	Adjusted *P* value
Age (years)					
≤40	106 (60.6)	69 (39.4)	1.07 (0.6–2.0)	1.06 (0.5–2.1)	0.86
>40	36 (62.1)	22 (37.9)	1	1	
Gender					
Female	65 (58.0)	47 (42.0)	1.26 (0.8–2.1)	1.26 (0.7–2.3)	0.43
Male	77 (63.6)	44 (36.8)	1	1	
Facility					
Public	23 (38.3)	37 (61.7)	3.6 (1.9–6.5)	3.8 (1.9–7.7)	<0.001
Private	119 (68.8)	54 (31.2)	1	1	
Treatment category					
New	132 (60.8)	85 (39.2)	1.07 (0.4–3.1)	1.06 (0.4–3.2)	0.92
Previously treated	10 (62.5)	6 (37.5)	1	1	
Type of TB					
Pulmonary	139 (61.8)	86 (38.2)	1	1	
Extrapulmonary	3 (37.5)	5 (62.5)	2.7 (0.6–11.6)	1.2 (0.3–6.0)	0.80
Anti-TB regimen					
Regimen 1	72 (55.8)	57 (44.2)	1.6 (0.9–2.8)	1.7 (0.9–3.2)	0.096
Regimen 2	70 (67.3)	34 (32.7)	1	1	
Received ART					
Yes	47 (58.8)	33 (41.2)	1.2 (0.7–2.0)	1.2 (0.5–2.5)	0.71
No	95 (62.1)	58 (37.9)	1	1	
Received CPT					
Yes	79 (60.3)	52 (39.7)	1.06 (0.6–1.8)	1.0 (0.5–2.0)	0.98
No	63 (61.8)	39 (38.2)	1	1	

Regimen 1: 2RHZE/6EH; regimen 2: 2RHZE/4RH (R: rifampicin, H: isoniazid, Z: pyrazinamide, and E: ethambutol); OR: odds ratio; ART: antiretroviral therapy; TB: tuberculosis; HIV: human immunodeficiency virus; CPT: cotrimoxazole preventive therapy.

**Table 3:** Predictors of unsuccessful tuberculosis treatment outcomes among 109 urban TB/HIV patients in Ebonyi State, Nigeria, 2011-2012.

Characteristics	Successful outcome n (%)	Unsuccessful outcome n (%)	Crude OR (95% CI)	Adjusted OR (95% CI)	Adjusted *P* value
Age (years)					
≤40	63 (78.8)	17 (21.2)	1	1	
>40	20 (69.0)	9 (31.0)	1.7 (0.6–4.3)	1.4 (0.5–3.8)	0.56
Gender					
Female	40 (76.9)	12 (23.1)	1	1	
Male	43 (75.4)	14 (24.6)	1.08 (0.5–2.6)	1.3 (0.5–3.5)	0.56
Facility					
Public	21 (67.7)	10 (32.3)	1.9 (0.7–4.7)	4.5 (1.2–16.7)	0.023
Private	62 (79.5)	16 (20.5)	1	1	
Treatment category					
New	78 (76.5)	24 (23.5)	1	1	
Previously treated	5 (71.4)	2 (28.6)	1.3 (0.2–7.1)	1.8 (0.3–11.1)	0.54
Type of TB					
Pulmonary	80 (75.5)	26 (24.5)			
Extrapulmonary	3 (100.0)	0.0	—	—	—
Anti-TB regimen					
Regimen 1	50 (74.6)	17 (25.4)	1.3 (0.5–3.1)	1.2 (0.4–3.7)	0.74
Regimen 2	33 (78.6)	9 (21.4)	1	1	
Received ART					
Yes	31 (83.8)	6 (16.2)	1	1	
No	52 (72.2)	20 (27.8)	2.0 (0.7–5.5)	7.0 (1.3–36.8)	0.021
Received CPT					
Yes	45 (77.6)	13 (22.4)	1	1	
No	38 (74.5)	13 (25.5)	1.2 (0.5–2.9)	1.4 (0.5–4.4)	0.53

Regimen 1: 2RHZE/6EH; regimen 2: 2RHZE/4RH (R: rifampicin, H: isoniazid, Z: pyrazinamide, and E: ethambutol); OR: odds ratio; ART: antiretroviral therapy; TB: tuberculosis; HIV: human immunodeficiency virus; CPT: cotrimoxazole preventive therapy.
